# Spontaneous infection of a stable mediastinal cystic mass: A case report

**DOI:** 10.1186/1757-1626-1-126

**Published:** 2008-08-26

**Authors:** Aldoph B Nanguzgambo, Martin Pike, Richard D Page, Grant F Benfield, Damian McKeon

**Affiliations:** 1 Department of Respiratory Medicine, Gwynedd Hospital, Bangor, UK; 2 Department of Radiology, Countess of Chester Hospital, Chester, UK; 3 Department of Cardiothoracic Surgery, Cardiothoracic Centre, Liverpoool, UK

## Abstract

Mediastinal cysts have an unpredictable course but can cause complications such as infection or local pressure effects. Persons with mediastinal cysts can be asymptomatic for many years or can develop symptoms as a result of complications of the cyst. There is a lack of consensus on the best approach to managing those patients without symptoms. In this case report, a 56 year old woman with an indolent mediastinal cyst initially managed conservatively suddenly developed symptoms suggestive of an infected mediastinal cyst requiring surgical resection.

## Introduction

Mediastinal cysts comprise 10–18% of radiologically detected masses in the mediastinum [1,2]. Foregut cysts including bronchogenic cysts are the most common and comprise 50% of all mediastinal cysts [2,3]. Complications of mediastinal cysts include infection (in about 30–36%), local pressure effects and malignant transformation [2-4]. The frequency of symptoms due to mediastinal cysts ranges from 35–90% and these include chest pain, dyspnoea, wheeze, cough, fever and hoarsenes of voice [2,4,5]. There is a lack of consensus on the best approach to managing those patients without symptoms. We present a case of a simple mediastinal cyst that was initially managed conservatively.

## Case

**Figure 1:**
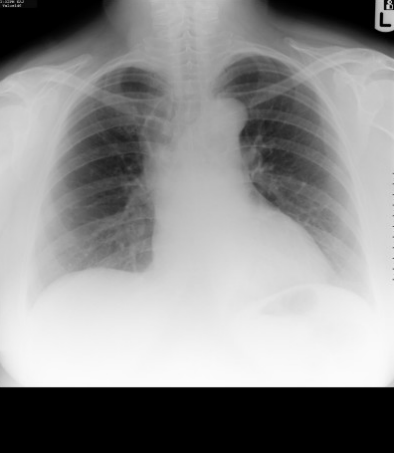
Chest radiograph showing the air-filled mediastinal mass.

**Figure 2:**
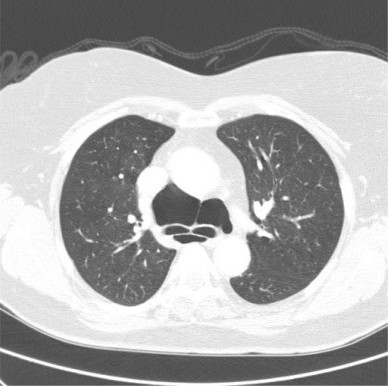
Contrast enhanced axial CT-scan of the thorax showing the air-filled cystic mass both anterior and posterior to the carina (lung window setting).

A 56 year old previously healthy Caucasian woman and non-smoker, presented to the outpatient chest department with symptoms of episodic bouts of dry cough associated with an occasional wheeze for 12 months and upper thoracic back pain for 3 months. She had no history of chest trauma. On examination, she was not breathless or wheezy and she had a normal temperature. The chest radiograph revealed an area of gas-filled tissue in the upper right mediastinum (fig. 1) and a Computer Tomography (CT) scan confirmed a loculated air-filled collection predominantly anterior to the trachea and extending below the carina with no evidence of fluid within the locules. There was no air tracking into the neck or the abdomen. (fig. 2). The patient had no recollection of having the cyst diagnosed in the past. The patient was stable and a wait and watch approach was taken, and the patient was to be reviewed in 3 months.

**Figure 3:**
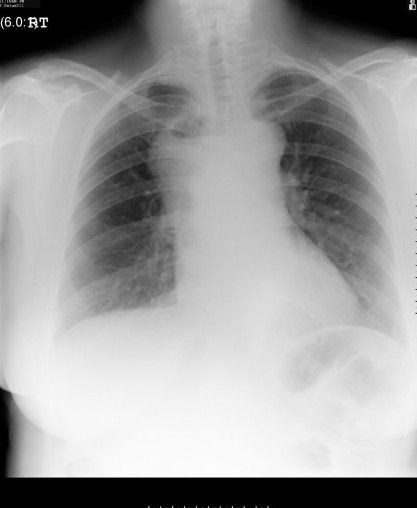
Chest radiograph showing the cystic mass, now with an air-fluid level (compare Fig 1).

**Figure 4:**
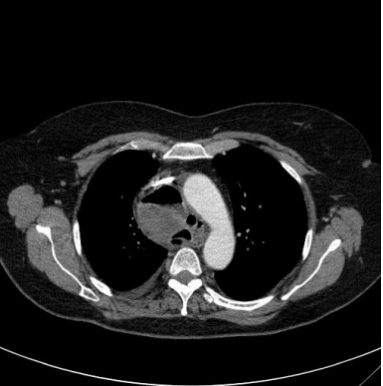
Contrast enhanced axial CT-scan of the thorax showing the cystic mass, now containing fluid, at the level of the aortic arch (mediastinal window setting).

**Figure 5:**
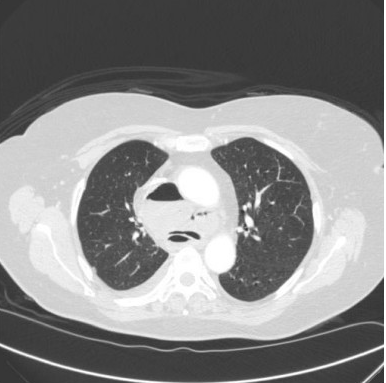
**Contrast enhanced axial CT-scan of the thorax showing the cystic mass at the level of the carina, now with air-fluid levels, compare fig 2**. Also shows bilateral pleural effusions worse on right side (lung window setting).

She was readmitted 2 months later with severe chest pain. On examination she was breathless with an expiratory wheeze and was febrile (37.5°C). The patient was not in shock. A repeat chest radiograph (fig 3) and CT scan (figs. 4 and 5) demonstrated a 9 × 5 cm loculated mass containing fluid and gas, encasing the lower trachea and the main proximal bronchi and extending from the innominate vein to the left atrium consistent with a mediastinal abscess. Bilateral pleural effusions were also present. The lungs were normal. The patient was treated with broad spectrum antibiotics and then had an open thoracotomy three days later with complete resection of the mass. The air-fluid level seen on the CT scans would suggest a tracheobronchial communication but no such communication was found during the operation. Histopathological examination of the mass revealed a collapsed thick walled cyst about 55 mm in diameter. The cyst wall consisted of fibrous and granulation tissue with heavy, chronic active inflammation. There were fragmented seromucinous glands on the inner surface of the cyst wall. These findings were consistent with an infected cyst likely to be bronchogenic in origin. Culture results of the cystic contents were however negative. The patient recovered from the surgical operation uneventfully and had no recurrence of her previous symptoms when reviewed 3 months later

## Discussion

Our patient first presented to us when she already had symptoms. It is not clear how long she had been with the cyst. We can only assume that it could have been congenital. We can not explain the subsequent development of the pleural effusions, nor the lack of microbial growth in the cystic contents after resection. We however postulate that the pleural effusions could have been reactive and the lack of any microbial growth could be due to the pre-operative antibiotics she received. We acknowledge that we did not aspirate fluid from the pleural effusions for analysis.

Initially we chose to manage her by observation. At the time of first presentation, she had symptoms of cough, wheeze and thoracic back pain which can be attributed to compression by the cyst on the tracheobronchial tree. This should have been the time to refer her to the thoracic surgeons for further management. Our conservative approach did not work and the patient susbsequently developed signs of an infected cyst that eventually required radical treatment. The current available treatment options for bronchogenic cysts, whether asymptomatic or symptomatic, include observation, fine needle aspiration, mediastinoscopic aspiration and biopsy, thoracoscopy, and mediastinoscopy or thoracotomy for resection [6]. Some authors advocate a conservative approach for small classic asymptomatic cysts [6-8]. Conservative management is defined by observation or minimal invasive procedures rather than open thoracotomy. The argument given is the lack of long term follow-up data on asymptomatic patients to know the natural history of the cysts, a very low risk of malignant transformation, the improved diagnostic radiological methods (CT scanning and MRI) and better diagnostic tools (thoracoscopy or transbroncial needle aspiration or mediastinoscopic aspiration) now available that can help avoid unnecessary surgical thoracotomies [6,7]. We are not sure whether such aspiration would have been the appropriate course for our patient at initial presentation to relieve her symptoms.

Other authors however advocate a radical approach by complete resection of the cyst using an open thoracotomy [2,4,9,10]. The reasons forwarded for radical resection are threefold. Firstly, complete resection prevents future complications. Although the natural history of bronchogenic cysts is not known, majority of the cysts will eventually cause symptoms. In the study by St Georges et al, 43% of the patients eventually became symptomatic although they had been known to have the mediastinal cysts for periods ranging from 6 months to several years [4]. In the series by Patel et al, 3 patients followed up for periods between 1.5 to 10 years eventually required resection due to development of symptoms [10]. Secondly, despite the low risk of malignant transformation of the cyst, resection helps to definitely exclude malignancy. Resection also provides a definite diagnosis as to the nature of the lesion. Although CT scanning can outline the lesion & define the contents (low hounsefield units), the density of the cyst may vary and make precise diagnosis by imaging difficult [6,9]. CT scanning only correctly defined the benign cystic nature of 5 lesions out of 8 in one series (62.5%) [10]. St Georges and colleagues have also discouraged aspiration of the cystic contents as a diagnostic or therapeutic procedure because the aspirate does not provide specific data on the cyst epithelium, the aspirate may be insufficient to exclude malignancy, and needling the cyst may predispose it to being infected. Thirdly, complete resection of symptomatic mediastinal cysts seems to be associated with greater intra- or post-operative complications than performing surgery in asymptomatic patients. In the series by Patel et al, there was a trend towards increased post-operative complications in those operated at time of symptom presentation compared to asymptomatic patients (27% vs 14%) [10]. It is also noted that about 44% of patients with mediastinal bronchogenic cysts in the study by St Georges et al had major operative difficulties or intra-operative complications and all of them were symptomatic [4].

Despite the lack of consensus on how to manage asymptomatic patients, there seems to be a general agreement that when the bronchogenic cyst increases in size or causes symptoms, intervention is warranted. Patients diagnosed with bronchogenic cysts should be referred to thoracic surgeons early so that the treatment options are explained to them to enable them make an informed choice on what intervention is appropriate at the time. However, the type of intervention will ultimately depend on the operating surgeon.

## Consent section

Written informed consent was obtained from the patient for the publication of this case report and accompanying images. A copy of the written consent is available for review by the Editor-in-Chief of this journal.

## Competing interests

The authors declare that they have no competing interests.

## Authors' contributions

ABN coordinated and wrote the manuscript. MP reported, prepared and labelled the radiology images. RDP contributed to the surgical aspects of the case. GFB and DM suggested the writing up of this case and reviewed the manuscript. All authors read and approved the manuscript.
